# A Machine Learning Sepsis Prediction Algorithm for Intended Intensive Care Unit Use (NAVOY Sepsis): Proof-of-Concept Study

**DOI:** 10.2196/28000

**Published:** 2021-09-30

**Authors:** Inger Persson, Andreas Östling, Martin Arlbrandt, Joakim Söderberg, David Becedas

**Affiliations:** 1 Department of Statistics Uppsala University Uppsala Sweden; 2 AlgoDx AB Stockholm Sweden; 3 Department of Anaesthesiology and Intensive Care Södersjukhuset Stockholm Sweden

**Keywords:** sepsis, prediction, early detection, machine learning, electronic health record, EHR, software as a medical device, algorithm, detection, intensive care unit, ICU, proof of concept

## Abstract

**Background:**

Despite decades of research, sepsis remains a leading cause of mortality and morbidity in intensive care units worldwide. The key to effective management and patient outcome is early detection, for which no prospectively validated machine learning prediction algorithm is currently available for clinical use in Europe.

**Objective:**

We aimed to develop a high-performance machine learning sepsis prediction algorithm based on routinely collected intensive care unit data, designed to be implemented in European intensive care units.

**Methods:**

The machine learning algorithm was developed using convolutional neural networks, based on Massachusetts Institute of Technology Lab for Computational Physiology MIMIC-III clinical data from intensive care unit patients aged 18 years or older. The model uses 20 variables to produce hourly predictions of onset of sepsis, defined by international Sepsis-3 criteria. Predictive performance was externally validated using hold-out test data.

**Results:**

The algorithm—NAVOY Sepsis—uses 4 hours of input and can identify patients with high risk of developing sepsis, with high performance (area under the receiver operating characteristics curve 0.90; area under the precision-recall curve 0.62) for predictions up to 3 hours before sepsis onset.

**Conclusions:**

The prediction performance of NAVOY Sepsis was superior to that of existing sepsis early warning scoring systems and comparable with those of other prediction algorithms designed to predict sepsis onset. The algorithm has excellent predictive properties and uses variables that are routinely collected in intensive care units.

## Introduction

Sepsis is a life-threatening clinical syndrome caused by dysregulated host response to infection [1]. Sepsis and the inflammatory response that ensues can lead to multiple organ dysfunction syndrome and death. It has been estimated that sepsis is present in 6% of adult hospital admissions [2] and in approximately one-third of intensive care unit (ICU) patients [3]. Globally, it affects approximately 49 million people every year [4]. During the coronavirus disease 2019 (COVID-19) pandemic, sepsis was the most frequently observed complication among adult inpatients at Jinyintan Hospital and Wuhan Pulmonary Hospital (Wuhan, China) who had been discharged or had died (as of January 31, 2020) [5].

There is a continuum of severity, ranging from sepsis to septic shock. Although wide-ranging and dependent upon study populations, mortality has been estimated to be at least 10%, and at least 40% when septic shock is present [1]. Despite decades of research, sepsis remains a leading cause of mortality and morbidity in modern ICUs worldwide [3]. The World Health Assembly and World Health Organization made sepsis a global health priority in 2017 and adopted a resolution to improve the prevention, diagnosis, and management of sepsis.

Early detection and effective management of sepsis is crucial, especially in ICUs—where the most critically ill patients are treated. Early diagnosis of sepsis has been shown to reduce delays in treatment, increase appropriate care, and reduce mortality [6-9]. A retrospective analysis of 17,000 patients has shown that there is a linear increase in the risk of mortality for each hour of delay in antibiotic administration [10]. Although sepsis is a potentially fatal condition, there is general consensus in guidelines [11] that early and relatively inexpensive intervention with antibiotics, fluid resuscitation, source control, and support of vital organ function lead to dramatically improved patient outcomes.

Early recognition of sepsis can be difficult due to its syndromic nature and patient heterogeneity. Early recognition is further complicated by the lack of reliable blood- or plasma-based biomarkers. Hundreds of biomarkers have been tested as prognostic markers in sepsis [12-14]; however, none has demonstrated sufficient specificity or sensitivity to be routinely used in clinical practice [12]. In this context, there exists a significant unmet medical need to assist clinicians with identifying hospitalized patients at risk of developing sepsis.

Today, sepsis diagnosis is made by combining information from clinical examinations performed by health care professionals and information provided from monitoring devices and laboratory data (ie, based on empirical clinical decision rules). This procedure is both time-consuming and subjective (ie, heavily dependent upon the skills and experience of the doctor or nurse). Timely intervention is critical for patients with sepsis, yet with the manual routines used at present, there is a risk of delayed diagnosis of sepsis and initiation of treatment.

Given that ICU clinicians are inundated with ever-increasing amounts of data collected at higher and higher resolution, machine learning prediction algorithms have gained increased interest in research and clinical practice because of their potential to improve early detection and adherence to treatment protocols and decrease time to antibiotic administration, which have been proved to improve clinical outcomes [6-9].

Fleuren et al [15] and Moor et al [16] reviewed previously developed sepsis prediction algorithms; however, they found that very few had been prospectively evaluated in clinical practice, and those that had been, were evaluated in the United States to date. To date, and to the best of our knowledge, only 1 ICU algorithm is available for clinical use [17,18], and another is planned to be prospectively validated [19].

The purpose of this proof-of-concept study was to develop a machine learning algorithm for early prediction of which patients in ICUs will develop sepsis within coming hours, using clinical data routinely collected in electronic health records.

## Methods

### Data Set and Study Population

The algorithm for prediction of sepsis was developed based on Massachusetts Institute of Technology Lab for Computational Physiology MIMIC-III Clinical Database [20]. This database contains demographic, vital sign, laboratory test, medication, and other data for 38,597 adult ICU patients (61,532 ICU stays), for whom data were collected between 2001 and 2012. At the time of algorithm development, the newer MIMIC-IV data were not available.

Sepsis was defined by Sepsis-3 criteria [1], which require a suspected infection and an increase in Sequential Organ Failure Assessment score of at least 2 points. Suspected infection [21] was defined as instances when antibiotics had been prescribed and when body fluid cultures were present in the electronic health record within a specific time window; if a culture is ordered within 24 hours after antibiotics, or antibiotics had been prescribed less than 72 hours after a culture order, the time of suspected infection was determined to be the earlier of these two. Sepsis-relevant antibiotics and body fluids were chosen as the indicators based on methods used by Liu et al [22]—they used blood cultures and a defined list of antibiotics (presented in the code repository referred to in their paper). A patient was considered septic if their Sequential Organ Failure Assessment score had increased by at least 2 points within the time window from 48 hours before to 24 hours after the time of suspected infection, and the time of sepsis onset was defined as the time of the 2-point increase. All patients not fulfilling Sepsis-3 criteria were defined as the nonsepsis cohort. The code used for assigning sepsis labels is available upon request.

**Figure 1:**
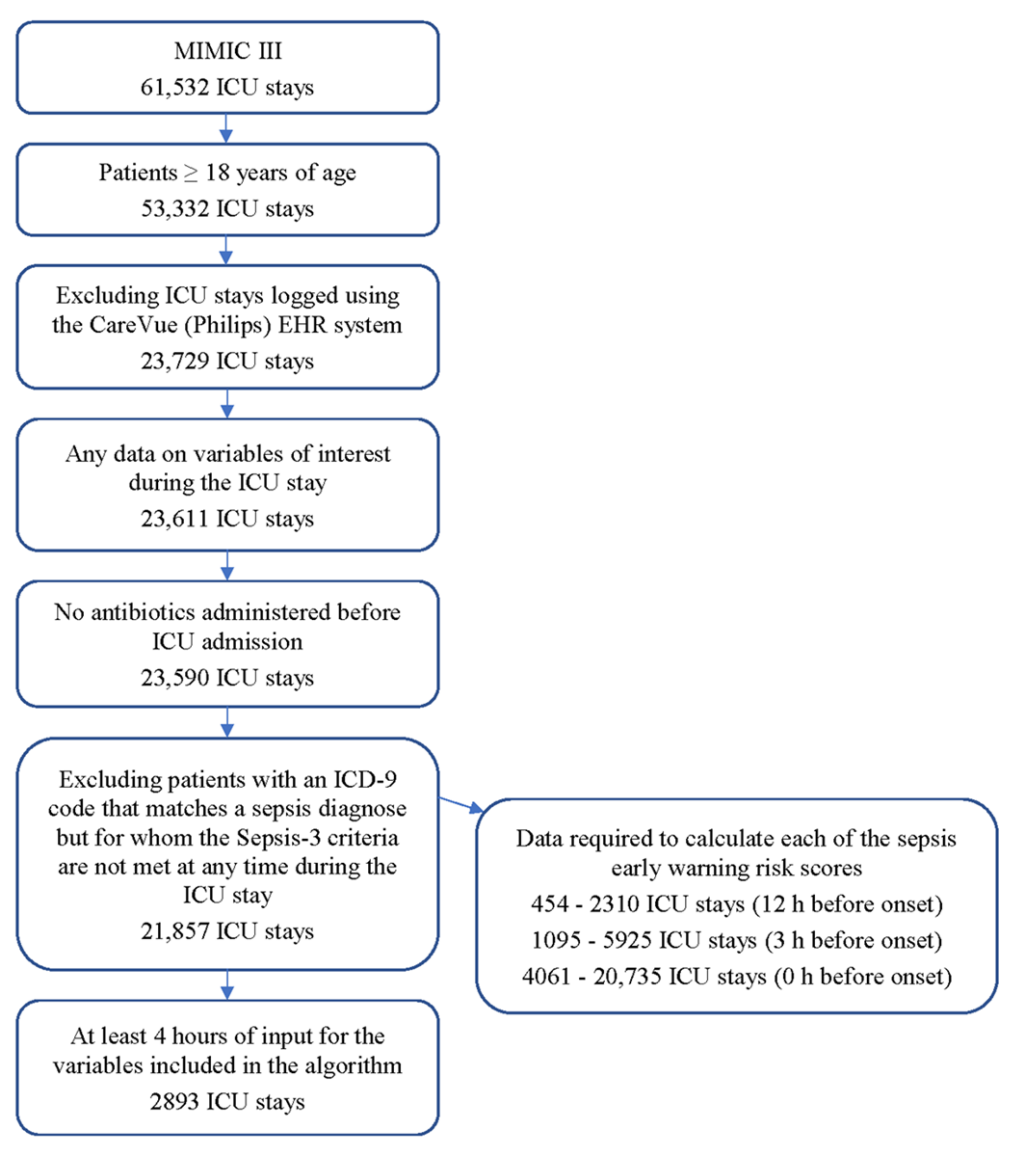
Intensive care unit (ICU) stays included in the analyses. EHR: electronic health record; *ICD-9*: *International Statistical Classification of Disease, ninth revision*.

Patients included (Figure 1; Table 1) in the analysis had at least 1 measurement of each of the variables included in the algorithm and were at least 18 years of age at the time of admission. Patients receiving antibiotics before ICU admission and patients with an *International Statistical Classification of Diseases, ninth revision*, (*ICD-9*) code that matched a sepsis diagnosis but for whom Sepsis-3 criteria were not met at any time during the ICU stay were not included. The latter can occur, for example, when the patient already had received a sepsis diagnosis at admission. No time stamps are available; therefore, diagnosis cannot be confirmed retrospectively. Differences between sepsis and nonsepsis cohorts were assessed using an appropriate test of statistical significance (Welch *t* test for numerical variables; the Fisher exact test or chi-square test for categorical variables). ICU stays logged using the CareVue (Philips) electronic health record system were excluded, that is, only ICU stays logged using the Metavision (iMDSoft) electronic health record system were included, since negative blood cultures are underreported with CareVue, which means that suspicion of infection is underrepresented in these patients [17].

The algorithm used the following 20 variables: age, gender, heart rate, respiratory rate, temperature, systolic blood pressure, diastolic blood pressure, vasopressor use, serum creatinine, glucose, lactate, platelets, white blood cell count, blood urea nitrogen, bilirubin, pH, oxygen saturation pulse oximetry, fraction of inspired oxygen, International Normalized Ratio, and Glasgow Coma Scale. Hourly values were used, and a last observation carried forward approach was used for single missing data points. For any hours with more than 1 measurement, hourly averages were used. Variable selection for the algorithm was conducted in cooperation with medical professionals to ensure that spurious variables were excluded and the most important variables were included. Any additional feature engineering was deemed unnecessary and was left for the network to discover.

**Table 1:** Characteristics of the patient population used for algorithm development and validation.

Patient characteristic			Sepsis		Nonsepsis		*P* value
**Age (years)**							
	Mean (SD)	60.9 (16.3)		64.7 (15.7)		<.001	
	Median (IQR)	61.6 (49.7-73.7)		66.2 (54.6-76.8)			
**Age groups (years), n (%)**							.002
	18-29	18.0 (4.4)		78.0 (3.1)			
	30-39	20.0 (4.9)		84.0 (3.4)			
	40-49	57.0 (14.1)		228.0 (9.2)			
	50-59	79.0 (19.5)		441.0 (17.7)			
	60-69	9.0 (2.2)		49.0 (2.0)			
	≥70	222.0 (54.8)		1608.0 (64.6)			
**Gender, n (%)**							.08
	Female	153.0 (37.8)		1055.0 (42.4)			
	Male	252.0 (62.2)		1433.0 (57.6)			
**Length of ICU^a^ stay (days)**							
	Mean (SD)	12.2 (9.4)		6.1 (5.9)		<.001	
	Median (IQR)	10.9 (5.0-16.6)		4.1 (2.3-7.7)			
**Length of ICU stay (days), n (%)**							<.001
	0-4	89.0 (22.0)		1344.0 (54.0)			
	5-9	96.0 (23.7)		692.0 (27.8)			
	10-14	78.0 (19.3)		260.0 (10.5)			
	15-19	70.0 (17.3)		93.0 (3.7)			
	20-24	30.0 (7.4)		50.0 (2.0)			
	25+	42.0 (10.4)		49.0 (2.0)			
**Time from ICU admission to sepsis onset (hours)**							N/A^b^
	Mean (SD)	32.0 (57.9)		N/A			
	Median (IQR)	4.3 (0.9-36.6)		N/A			
**Antibiotics administered before or at time of sepsis onset, n (%)**							N/A
	Yes	4.0 (1.0)		N/A			
	No	401.0 (99.0)		N/A			
**Comorbidities^c^, n (%)**							
	Renal disease	235.0 (58.0)		1070.0 (43.0)		<.001	
	Diabetes	117.0 (28.9)		750.0 (30.1)		.64	
	Respiratory disease	370.0 (91.4)		1709.0 (68.7)		<.001	
	Cardiovascular disease	364.0 (89.9)		2252.0 (90.5)		.72	
	Liver disease	110.0 (27.2)		468.0 (18.8)		<.001	
	Cancer	71.0 (17.5)		462.0 (18.6)		.68	
**Admission to type of intensive care unit, n (%)**							<.001
	Medical intensive care unit	191.0 (47.2)		771.0 (31.0)			
	Cardiac surgery recovery unit	43.0 (10.6)		619.0 (24.9)			
	Surgical intensive care unit	70.0 (17.3)		493.0 (19.8)			
	Trauma Surgical intensive care unit	58.0 (14.3)		303.0 (12.2)			
	Coronary care unit	43.0 (10.6)		302.0 (12.1)			
**Death during hospital stay, n (%)**							<.001
	Yes	118.0 (29.1)		328.0 (13.2)			
	No	287.0 (70.9)		2160.0 (86.8)			

^a^ICU: intensive care unit.

^b^N/A: not applicable.

^c^Comorbidities are defined by *International Statistical Classification of Diseases, ninth revision*, codes recorded during the intensive care unit stay.

### Machine Learning Algorithm Development

The algorithm was developed using convolutional neural networks [23]. This method was chosen based on its ability to handle time series data. Data were preprocessed using R (The R Project), and the models were executed using TensorFlow [24,25] backend in Python (version 3.7.6) via Jupyter Notebooks (version 6.0.3).

The model has 2 convolutional layers, the first with 10 filters, the second with 5 filters, each of size (1,2) where 1 is the variable domain and 2 is the time domain. The filter walks across the variables one by one, looking at each pair of time points for that variable. The convolutional layers are followed by 4 fully connected layers of size 50, 25, 15, 10, respectively, before feeding into the final output layer. Dropout with parameter 0.5 is performed between each layer, both convolutional and fully connected.

The batch size for training was 512. Training continued until the training loss had not improved in the last 1000 epochs (early stopping), after which the weights with the lowest training loss were saved. A cyclical learning rate was used [26] (with initial learning rate: 1e–4, maximal learning rate: 1e–3, step size: 16 * number of training examples).

Different parts of the training data were used for development and internal validation of the algorithm in order to avoid overfitting. Random onset matching [16]—randomly chosen 4-hour sequences, with the last time point up to 3 hours before onset, for patients with sepsis, or at any point during the whole ICU stay, for patients without sepsis—was used. The time points were sampled from a β(10,1) distribution, with ranges for patients without sepsis scaled to match those of their entire stay. The β parameters were chosen to place higher weights early in their stay. Data were sampled to maintain a prevalence of sepsis of 20% in both training and test data, to resemble the prevalence of sepsis in ICU patients in North America and Western Europe [3]. This also facilitated comparisons between training and test data, since area under the receiver operating characteristic curve (AUROC), area under the precision-recall curve (AUPRC), and accuracy are affected by prevalence. A prediction horizon of 3 hours was chosen based on the availability of data at different time points; at earlier time points, there were considerably fewer ICU stays with data for all variables of interest. In a similar study, data imputation was performed for early time points with missing data, for example, by copying the first available data to earlier time points [17]; however, this technique would be impossible to use in a live setting; thus, it was not used in our study. The training data consisted of 7681 sequences (n=2593 ICU stays) of 4-hour data (sepsis: n=1385 sequences, nonsepsis: n=6296 sequences), and internal validation during training was performed on 633 sequences of 4-hour data (n=200 ICU stays). The final algorithm was externally validated using the second part of the data (hold-out test data, ie, data that were not used in development of the algorithm; n=95 ICU stays, n=152 sequences of 4-hour data).

### Comparison With the Predictive Abilities of Related Scores

Performance of the algorithm was compared with a number of illness severity risk scores currently used in clinical practice to predict sepsis in the same time frame (for a summary of sepsis early warning scoring systems, see Postelnicu et al [27] and Rosenqvist [28]). The following scores were included in this study: Systemic Inflammatory Response Syndrome criteria, at least 2 of 4 criteria present [29]; Quick Sepsis-Related Organ Failure Assessment score, at least 2 of 3 criteria present [1]; Sepsis-Related Organ Failure Assessment score, total score ≥2 [1]; Modified Early Warning Score, score ≥5 [30]; National Early Warning Score 2, score ≥ 5 [31]; Rapid Emergency Triage and Treatment System, highest priority level [32];Sepsis Alert [28]; and Prehospital Early Sepsis Detection score, score ≥4 [33]. Predictions were computed at the same time points as those of the algorithm.

### Performance

Receiver operating characteristics, that is, the proportions of true positives (sensitivity) relative to the proportions of false positives (1 – specificity), were calculated to assess performance. Based on the receiver operating characteristics curve, an operating point (threshold) was chosen for classification of patients with high risk of developing sepsis. True positives were patients with sepsis that were accurately identified by the algorithm up to 3 hours before the onset of sepsis, and false positives were patients without sepsis that were incorrectly identified by the algorithm to be at risk of developing sepsis. The operating point for the algorithm was chosen to keep a sensitivity (proportion of true positives) of approximately 0.80 and a higher specificity (proportion of true negatives), in order to minimize the false alert rate while still keeping a high sensitivity. Ideally, an algorithm should yield a high proportion of true positives and a low proportion of false positives, which corresponds to a large AUROC. The AUPRC is also of importance—a large area represents both high recall (low false negative rate) and high precision (low false positive rate). High scores for both recall and precision demonstrate accurate results (high precision) and mostly positive results (high recall). Accuracy is the proportion of correct predictions. Positive predictive value is the proportion of predicted sepsis cases that are true sepsis cases). Further information about accuracy, sensitivity, and specificity can be found in Multimedia Appendix 1.

## Results

The AUROC for the algorithm was as high as 0.90 on internally validated training data (Table 2) and 0.84 on hold-out test data, for predictions 3 hours before onset (Table 3). The AUPRC was 0.62 on training data (Table 2) and as high as 0.68 on test data, for predictions 3 hours before onset (Table 3).

**Figure 2:**
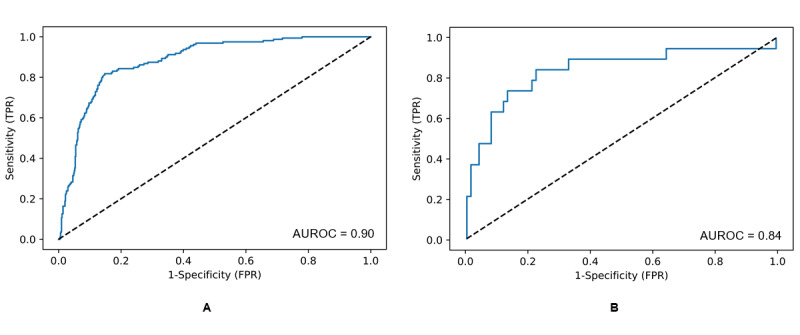
Receiver operating characteristics curve of the algorithm for (A) training data and (B) hold-out test data predictions 3 hours before sepsis onset. AUROC: area under the receiver operating characteristics curve; FPR: false positive rate; TPR: true positive rate.

**Figure 3:**
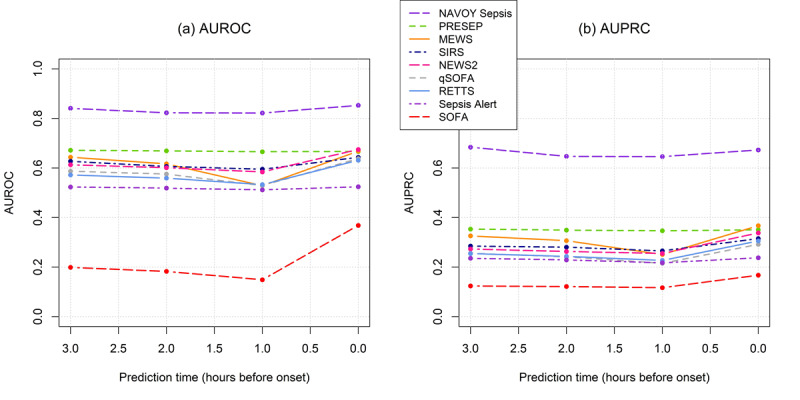
(A) AUROC and (B) AUPRC for comparison risk scores for sepsis predictions up to 3 hours in advance. AUPRC: area under the precision-recall curve; AUROC: area under the receiver operating characteristics curve; MEWS: Modified Early Warning Score; NEWS2: National Early Warning Score 2; PRESEP: Prehospital Early Sepsis Detection; qSOFA: Quick Sepsis-Related Organ Failure Assessment; RETTS: Rapid Emergency Triage and Treatment System; SIRS: Systemic Inflammatory Response Syndrome; SOFA: Sepsis-Related Organ Failure Assessment.

The algorithm’s sensitivity, specificity, and accuracy were higher than those for any of the comparison risk scores (Table 2, Table 3, Figure 2, and Figure 3). In external validation (Table 3), sensitivity values for predictions 3 hours before onset were higher than those at any of the time points closer to onset. This was expected, since NAVOY Sepsis was optimized to make predictions as early as possible. The operating point produced a positive predictive value of 0.57 on training data (Table 2), and 0.50 on test data, for predictions 3 hours before onset (Table 3). This metric was expected to be lower than sensitivity, specificity, and accuracy, due to the severe class imbalance. A sensitivity of 85% produces 15% false positives; since the majority of patients did not have sepsis, sepsis will be overpredicted. When comparing the distribution of sepsis predictions made by the algorithm with the actual distribution of sepsis (prevalence), the algorithm predicted that 28% of patients had sepsis in training data (Table 2) and 27% to 29% of patients had sepsis in test data (Table 3), which is somewhat larger than the prevalence of 20%.

**Table 2:** Internal validation performance (using training data) for algorithm predictions up to 3 hours in advance.

Performance metric	Value
AUROC^a^	0.90
AUPRC^b^	0.62
Accuracy (95% CI)^c^	0.86 (0.80, 0.91)
Sensitivity	0.80
Specificity	0.85
Positive predictive value	0.57
Proportion of predicted sepsis	0.28

^a^AUROC: area under the receiver operating characteristic curve.

^b^AUPRC: area under the precision-recall curve.

^c^Operating points for the algorithm were chosen to keep sensitivity at approximately 0.80.

**Table 3:** Performance on hold-out test data for algorithm predictions up to 3 hours in advance.

Performance metric	3 hours before onset	2 hours before onset	1 hour before onset	0 hours before onset
AUROC^a^	0.84	0.82	0.82	0.85
AUPRC^b^	0.68	0.67	0.65	0.67
Accuracy (95% CI)^c^	0.81 (0.73, 0.89)	0.79 (0.71, 0.87)	0.79 (0.71, 0.87)	0.79 (0.71, 0.87)
Sensitivity	0.74	0.63	0.63	0.63
Specificity	0.83	0.83	0.83	0.83
Positive predictive value	0.50	0.46	0.46	0.46
Proportion of predicted sepsis	0.29	0.27	0.27	0.27

^a^AUROC: area under the receiver operating characteristic curve.

^b^AUPRC: area under the precision-recall curve.

^c^Operating points for the algorithm were chosen during training and internal validation to keep sensitivity at approximately 0.80.

## Discussion

### Principal Results

Only 1% (4/405) of the patients with sepsis included in the data set had antibiotics administered before or at the time of sepsis onset, which confirms that there is a need for NAVOY Sepsis as an early detection system. Almost no patients had complete data, which is similar to clinical use situations. The algorithm was designed to be integrated with electronic health record systems primarily in Europe (CE marked as Software as a Medical Device) and is currently being evaluated in what is expected to be the largest prospective randomized clinical trial of a machine learning sepsis prediction algorithm to date (ClinicalTrials.gov; NCT04570618). The algorithm has excellent predictive properties, outperforms existing early warning scoring systems, and is comparable to previously published algorithms [17,34-36] designed to predict sepsis onset for ICU patients in accordance with the Sepsis-3 criteria. The algorithm uses 4 hours of input from routinely collected variables to make sepsis predictions. This means that only a few hours after ICU admission, the clinical staff can receive high-performance risk assessment for sepsis in adult patients.

### Comparison With Prior Work

Moor et al [16] point out that it can be difficult to compare studies due to measures such as AUROC or accuracy as they are directly affected by sepsis prevalence. In unbalanced situations, such as in the case of sepsis prediction, where the proportion of patients without sepsis is substantially larger than the proportion of patients with sepsis, the AUPRC should be reported. The AUPRC of NAVOY Sepsis is, to the best of our knowledge, substantially higher than that shown by any comparable sepsis prediction algorithm to date (ranging between 0.04 and 0.60) [17,34-36]. The algorithm provides accurate results (high precision) and returns a majority of all positive results (high recall).

The AUROC curve is high, which means that NAVOY Sepsis yields a high proportion of true positives and a low proportion of false positives. The AUROC of NAVOY Sepsis is higher than those of many sepsis early warning scoring systems, evaluated using the same data. The AUROC of NAVOY Sepsis is also higher than those of all previously published algorithms (ranging between and 0.74 and 0.85) [17,19,36-38] but one [39], noting, however, the abovementioned comparability issues. Only Futoma et al [35] used a comparable sepsis prevalence (21%), with other prevalences ranging between 6% and 9% (or not specified) [17,19,36-38]. Only 1 sepsis prediction model [39] had a higher AUROC (as high as 0.97; AUPRC not presented) than that of NAVOY Sepsis. Wickramaratne and Mahmud [39] state that their model “has an advantage over the traditional methods in terms of using new data to improve performance. Further, the model can include new features when they become available.” In other words, their model [39] seems to be a self-learning model, which would be the first of its kind if used in practice. The paper describes the technical aspects of their proposed model well but does not discuss how to implement the model into clinical practice [39]. Obtaining regulatory clearance in Europe, in the form of a CE mark, for self-learning software for use in health care is not an easy task. However, Wickramaratne and Mahmud’s algorithm [39], as many of the other previous attempts described in the literature [36-38], is based on a number of laboratory tests not routinely performed in European ICUs and would thus not be relevant for the European Union market. NAVOY Sepsis is based only on variables that are routinely measured in European ICUs and was developed in collaboration with medical professionals to ensure that it will be applicable to clinical practice.

### Limitations

This study has some limitations. First, the algorithm was developed using retrospective data and has not yet been evaluated prospectively. As Moor et al [16] wisely point out, “only the demonstration of favorable outcomes in large prospective randomized controlled trials will pave the way for machine learning models entering the clinical routine.” Second, even though matching of sepsis onset time for patients without sepsis was used in order to prevent bias caused by differences in the length of stay distribution, other types of bias might be present. For example, performance metrics were affected by the prevalence of sepsis, and even though the prevalence was set at 20% to enable direct comparisons with early warning scores, it is difficult to compare our findings with those in previously published research. Third, it would have been valuable to test the performance of the algorithm with an additional external validation cohort, for example, data from the PhysioNet Challenge [38] or the eICU Collaborative Research Database [40]. However, the PhysioNet Challenge data do not contain all the variables of interest, and the eICU data only contain a few patients with information on all of the variables and thus could not be used for this purpose. It should, however, be noted that external validation was performed in this study (on hold-out test data). Fourth, this study does not provide information on the clinical or economic impact of the integration of the developed algorithm in clinical practice.

### Future Work

The accuracy, sensitivity, and specificity of the algorithm developed in this proof-of-concept study are to potentially be validated in a prospective randomized clinical trial (ClinicalTrials.gov; NCT04570618). That study also intends to further explore the developed algorithm’s integration into clinical workflow and effect on relevant clinical outcomes. In addition, a health economic study is currently being undertaken where the cost-effectiveness of implementation of the developed algorithm in European ICUs is being explored. Finally, when deploying the algorithm at different institutions, it will be important to evaluate its performance by, for example, using an initial period without presenting the predictions, to allow for a comparison of the predictions and sepsis onset and thereby enable adjustment of the threshold to ensure that the algorithm will work as expected at each institution. Also, with access to data from different institutions, the algorithm can be retrained and continuously improved or adjusted to work well in different settings (regions, hospitals, populations).

### Conclusions

Sepsis remains a leading cause of mortality and morbidity in ICUs worldwide. Early detection is key to effective management and patient outcome, as there is no specific sepsis treatment available. We have developed a high-performance machine learning sepsis prediction algorithm that outperforms existing early warning scoring systems. The algorithm is based on variables routinely collected and readily available in electronic health records in ICUs of all categories and may provide an opportunity for enhanced patient monitoring, earlier detection of sepsis, and improved patient outcomes. If the findings in this study are validated in the upcoming prospective randomized clinical trial, this algorithm has the potential to be the first CE-marked sepsis prediction algorithm for commercial use in European ICUs.

## Appendix

Multimedia Appendix 1 Supplementary material.

## Abbreviations

AUPRC: area under the precision-recall curve
AUROC: area under the receiver operating characteristics curve
*ICD-9*: *International Statistical Classification of Diseases, ninth revision*
ICU: intensive care unit
