# Supplementary material for: A Machine Learning Sepsis Prediction Algorithm for Intended Intensive Care Unit Use (NAVOY Sepsis): Proof-of-Concept Study
Source: JMIR Form Res. 2021 Sep 30;5(9):e28000. doi: 10.2196/28000 (PMC8517825; doi:10.2196/28000)
Supplement: Multimedia Appendix 1 [file formative_v5i9e28000_app1.docx]

**Supplemental Table 1: Performance Metrics for SIRS Predicting Sepsis up to 3 Hours in Advance, All ICU Stays with Available Data**

| **SIRS** | 3h | 2h | 1h | 0h |
| --- | --- | --- | --- | --- |
| n | 5925 | 7790 | 15775 | 20735 |
| AUROC | 0.6271 | 0.6065 | 0.5951 | 0.6431 |
| AUPRC | 0.2848 | 0.2804 | 0.2654 | 0.3149 |
| Accuracy*^a^*  ( 95% CI) | 0.6738 (0.6616, 0.6857) | 0.6582 (0.6475, 0.6687) | 0.6542 (0.6467, 0.6616) | 0.6709 (0.6645, 0.6773) |
| Sensitivity | 0.4937 | 0.4499 | 0.4316 | 0.5166 |
| Specificity | 0.7188 | 0.7102 | 0.7099 | 0.7091 |
| PPV | 0.3050 | 0.2796 | 0.2711 | 0.3054 |

*^a^* Threshold for sepsis prediction (operating point): SIRS≥2

SIRS = Systemic Inflammatory Response Syndrome criteria

n = number of ICU stays with non-missing information at this time point

AUROC = area under the receiver operating characteristic curve

AUPRC = area under the precision-recall curve

CI = confidence interval

PPV = positive predictive value

**Supplemental Table 2: Performance Metrics for SIRS Predicting Sepsis Up to 3 Hours in Advance, Population for Algorithm Development and Validation**

| **SIRS** | 3h | 2h | 1h | 0h |
| --- | --- | --- | --- | --- |
| n | 1115 | 1205 | 1685 | 1700 |
| AUROC | 0.6129 | 0.6013 | 0.6044 | 0.6265 |
| AUPRC | 0.2656 | 0.2616 | 0.2740 | 0.2911 |
| Accuracy *^a^*  ( 95% CI) | 0.6117 (0.5823, 0.6404) | 0.5900 (0.5617, 0.6180) | 0.5941 (0.5702, 0.6176) | 0.6065 (0.5828, 0.6298) |
| Sensitivity | 0.5874 | 0.5809 | 0.5608 | 0.5912 |
| Specificity | 0.6177 | 0.5923 | 0.6024 | 0.6103 |
| PPV | 0.2775 | 0.2627 | 0.2607 | 0.2750 |

*^a^* Threshold for sepsis prediction (operating point): SIRS≥2

SIRS = Systemic Inflammatory Response Syndrome criteria

n = number of ICU stays with non-missing information at this time point

AUROC = area under the receiver operating characteristic curve

AUPRC = area under the precision-recall curve

CI = confidence interval

PPV = positive predictive value

**Supplemental Table 3: Performance Metrics for NEWS2 Predicting Sepsis Up to 3 Hours in Advance, All ICU Stays with Available Data**

| **NEWS2** | 3h | 2h | 1h | 0h |
| --- | --- | --- | --- | --- |
| n | 5550 | 7005 | 14480 | 20385 |
| AUROC | 0.6131 | 0.6014 | 0.5841 | 0.6748 |
| AUPRC | 0.2729 | 0.2630 | 0.2552 | 0.3380 |
| Accuracy *^a^*  ( 95% CI) | 0.6505 (0.6377, 0.663) | 0.6471 (0.6358, 0.6583) | 0.6426 (0.6347, 0.6504) | 0.6673 (0.6607, 0.6737) |
| Sensitivity | 0.4523 | 0.4511 | 0.4206 | 0.5607 |
| Specificity | 0.7000 | 0.6961 | 0.6981 | 0.6939 |
| PPV | 0.2737 | 0.2707 | 0.2583 | 0.3141 |

*^a^* Threshold for sepsis prediction (operating point): NEWS2≥5

NEWS2 = National Early Warning Score 2

n = number of ICU stays with non-missing information at this time point

AUROC = area under the receiver operating characteristic curve

AUPRC = area under the precision-recall curve

CI = confidence interval

PPV = positive predictive value

**Supplemental Table 4: Performance Metrics for NEWS2 Predicting Sepsis Up to 3 Hours in Advance, Population for Algorithm Development and Validation**

| **NEWS2** | 3h | 2h | 1h | 0h |
| --- | --- | --- | --- | --- |
| n | 1100 | 1170 | 1635 | 1700 |
| AUROC | 0.6019 | 0.6082 | 0.5596 | 0.6954 |
| AUPRC | 0.2655 | 0.2642 | 0.2562 | 0.3468 |
| Accuracy *^a^*  ( 95% CI) | 0.4827 (0.4528, 0.5127) | 0.4932 (0.4641, 0.5222) | 0.4722 (0.4477, 0.4967) | 0.5229 (0.4989, 0.5469) |
| Sensitivity | 0.6636 | 0.6838 | 0.6391 | 0.8118 |
| Specificity | 0.4375 | 0.4455 | 0.4304 | 0.4507 |
| PPV | 0.2278 | 0.2356 | 0.2191 | 0.2698 |

*^a^* Threshold for sepsis prediction (operating point): NEWS2≥5

NEWS2 = National Early Warning Score 2

n = number of ICU stays with non-missing information at this time point

AUROC = area under the receiver operating characteristic curve

AUPRC = area under the precision-recall curve

CI = confidence interval

PPV = positive predictive value

**Supplemental Table 5: Performance Metrics for MEWS Predicting Sepsis Up to 3 Hours in Advance, All ICU Stays with Available Data**

| **MEWS** | 3h | 2h | 1h | 0h |
| --- | --- | --- | --- | --- |
| n | 5535 | 6970 | 14360 | 20375 |
| AUROC | 0.6437 | 0.6169 | 0.5285 | 0.6681 |
| AUPRC | 0.3260 | 0.3067 | 0.2507 | 0.3671 |
| Accuracy *^a^*  ( 95% CI) | 0.7749  (0.7637, 0.7858) | 0.7651  (0.755, 0.775) | 0.7528  (0.7456, 0.7598) | 0.7750  (0.7692, 0.7807) |
| Sensitivity | 0.2611 | 0.2626 | 0.1950 | 0.3139 |
| Specificity | 0.9033 | 0.8908 | 0.8922 | 0.8903 |
| PPV | 0.4031 | 0.3754 | 0.3115 | 0.4169 |

*^a^* Threshold for sepsis prediction (operating point): MEWS≥5

MEWS = Modified Early Warning Score

n = number of ICU stays with non-missing information at this time point

AUROC = area under the receiver operating characteristic curve

AUPRC = area under the precision-recall curve

CI = confidence interval

PPV = positive predictive value

**Supplemental Table 6: Performance Metrics for MEWS Predicting Sepsis Up to 3 Hours in Advance, Population for Algorithm Development and Validation**

| **MEWS** | 3h | 2h | 1h | 0h |
| --- | --- | --- | --- | --- |
| n | 1100 | 1170 | 1635 | 1700 |
| AUROC | 0.6289 | 0.6211 | 0.5624 | 0.7021 |
| AUPRC | 0.2758 | 0.2836 | 0.2461 | 0.3581 |
| Accuracy *^a^*  ( 95% CI) | 0.6791 (0.6506, 0.7066) | 0.6718 (0.6441, 0.6987) | 0.6514 (0.6277, 0.6745) | 0.6847 (0.6620, 0.7068) |
| Sensitivity | 0.3864 | 0.4103 | 0.3700 | 0.5029 |
| Specificity | 0.7523 | 0.7372 | 0.7217 | 0.7301 |
| PPV | 0.2805 | 0.2807 | 0.2495 | 0.3178 |

*^a^* Threshold for sepsis prediction (operating point): MEWS≥5

MEWS = Modified Early Warning Score

n = number of ICU stays with non-missing information at this time point

AUROC = area under the receiver operating characteristic curve

AUPRC = area under the precision-recall curve

CI = confidence interval

PPV = positive predictive value

**Supplemental Table 7: Performance Metrics for qSOFA Predicting Sepsis Up to 3 Hours in Advance, All ICU Stays with Available Data**

| **qSOFA** | 3h | 2h | 1h | 0h |
| --- | --- | --- | --- | --- |
| n | 5515 | 6960 | 14350 | 20375 |
| AUROC | 0.5872 | 0.5755 | 0.5291 | 0.6377 |
| AUPRC | 0.2557 | 0.2417 | 0.2153 | 0.2919 |
| Accuracy *^a^*  ( 95% CI) | 0.7277  (0.7157, 0.7394) | 0.7279  (0.7173, 0.7383) | 0.7143 (0.7068, 0.7217) | 0.7377 (0.7316, 0.7437) |
| Sensitivity | 0.2267 | 0.2155 | 0.1714 | 0.8037 |
| Specificity | 0.8529 | 0.8560 | 0.8500 | 0.4187 |
| PPV | 0.2781 | 0.2722 | 0.2222 | 0.3283 |

*^a^* Threshold for sepsis prediction (operating point): qSOFA≥2

qSOFA = quick Sepsis-Related Organ Failure Assessment score

n = number of ICU stays with non-missing information at this time point

AUROC = area under the receiver operating characteristic curve

AUPRC = area under the precision-recall curve

CI = confidence interval

PPV = positive predictive value

**Supplemental Table 8: Performance Metrics for qSOFA Predicting Sepsis Up to 3 Hours in Advance, Population for Algorithm Development and Validation**

| **qSOFA** | 3h | 2h | 1h | 0h |
| --- | --- | --- | --- | --- |
| n | 1100 | 1170 | 1635 | 1700 |
| AUROC | 0.6121 | 0.6225 | 0.5440 | 0.6374 |
| AUPRC | 0.2567 | 0.2619 | 0.2340 | 0.2901 |
| Accuracy *^a^*  ( 95% CI) | 0.6691 (0.6404, 0.6969) | 0.6701 (0.6423, 0.6970) | 0.6410 (0.6172, 0.6643) | 0.6724 (0.6495, 0.6946) |
| Sensitivity | 0.3864 | 0.4188 | 0.3242 | 0.4265 |
| Specificity | 0.7398 | 0.7329 | 0.7202 | 0.7338 |
| PPV | 0.2707 | 0.2816 | 0.2246 | 0.2860 |

*^a^* Threshold for sepsis prediction (operating point): qSOFA≥2

qSOFA = quick Sepsis-Related Organ Failure Assessment score

n = number of ICU stays with non-missing information at this time point

AUROC = area under the receiver operating characteristic curve

AUPRC = area under the precision-recall curve

CI = confidence interval

PPV = positive predictive value

**Supplemental Table 9: Performance Metrics for SOFA Predicting Sepsis Up to 3 Hours in Advance, All ICU Stays with Available Data**

| **SOFA** | 3h | 2h | 1h | 0h |
| --- | --- | --- | --- | --- |
| n | 5820 | 8055 | 16195 | 20665 |
| AUROC | 0.1989 | 0.1827 | 0.1489 | 0.3682 |
| AUPRC | 0.1238 | 0.1214 | 0.1169 | 0.1673 |
| Accuracy *^a^*  ( 95% CI) | 0.1713  (0.1617, 0.1812) | 0.1613  (0.1533, 0.1695) | 0.1447  (0.1394, 0.1502) | 0.2511  (0.2452, 0.2571) |
| Sensitivity | 0.5945 | 0.5413 | 0.4616 | 1.0000 |
| Specificity | 0.0655 | 0.0663 | 0.0655 | 0.0639 |
| PPV | 0.1372 | 0.1266 | 0.1099 | 0.2108 |

*^a^* Threshold for sepsis prediction (operating point): SOFA≥2

SOFA = Sepsis-Related Organ Failure Assessment score

n = number of ICU stays with non-missing information at this time point

AUROC = area under the receiver operating characteristic curve

AUPRC = area under the precision-recall curve

CI = confidence interval

PPV = positive predictive value

**Supplemental Table 10: Performance Metrics for SOFA Predicting Sepsis Up to 3 Hours in Advance, Population for Algorithm Development and Validation**

| **SOFA** | 3h | 2h | 1h | 0h |
| --- | --- | --- | --- | --- |
| n | 895 | 1090 | 1685 | 1700 |
| AUROC | 0.4503 | 0.4204 | 0.3845 | 0.7468 |
| AUPRC | 0.2076 | 0.1883 | 0.1741 | 0.4271 |
| Accuracy *^a^*  ( 95% CI) | 0.2078 (0.1817, 0.2359) | 0.2009 (0.1775, 0.226) | 0.1887 (0.1703, 0.2082) | 0.2135 (0.2039, 0.2441) |
| Sensitivity | 0.9106 | 0.9037 | 0.8427 | 1.0000 |
| Specificity | 0.0321 | 0.0252 | 0.0252 | 0.0294 |
| PPV | 0.1904 | 0.1882 | 0.1777 | 0.2048 |

*^a^* Threshold for sepsis prediction (operating point): SOFA≥2

SOFA = Sepsis-Related Organ Failure Assessment score

n = number of ICU stays with non-missing information at this time point

AUROC = area under the receiver operating characteristic curve

AUPRC = area under the precision-recall curve

CI = confidence interval

PPV = positive predictive value

**Supplemental Table 11: Performance Metrics for RETTS Predicting Sepsis Up to 3 Hours in Advance, All ICU Stays with Available Data**

| **RETTS** | 3h | 2h | 1h | 0h |
| --- | --- | --- | --- | --- |
| n | 5530 | 6970 | 14370 | 20375 |
| AUROC | 0.5719 | 0.5591 | 0.5329 | 0.6313 |
| AUPRC | 0.2546 | 0.2432 | 0.2269 | 0.3057 |
| Accuracy *^a^*  ( 95% CI) | 0.6841  (0.6716, 0.6963) | 0.6796  (0.6685, 0.6906) | 0.6660  (0.6583, 0.6738) | 0.7055  (0.6992, 0.7118) |
| Sensitivity | 0.3788 | 0.3544 | 0.3048 | 0.4974 |
| Specificity | 0.7604 | 0.7609 | 0.7564 | 0.7576 |
| PPV | 0.2833 | 0.2704 | 0.2382 | 0.3390 |

*^a^* Threshold for sepsis prediction (operating point): RETTS red (highest level)

RETTS = Rapid Emergency Triage and Treatment System

n = number of ICU stays with non-missing information at this time point

AUROC = area under the receiver operating characteristic curve

AUPRC = area under the precision-recall curve

CI = confidence interval

PPV = positive predictive value

**Supplemental Table 12: Performance Metrics for RETTS Predicting Sepsis Up to 3 Hours in Advance, Population for Algorithm Development and Validation**

| **RETTS** | 3h | 2h | 1h | 0h |
| --- | --- | --- | --- | --- |
| N | 1100 | 1170 | 1635 | 1700 |
| AUROC | 0.6126 | 0.5709 | 0.5557 | 0.6779 |
| AUPRC | 0.2596 | 0.2385 | 0.2357 | 0.3134 |
| Accuracy *^a^*  ( 95% CI) | 0.5527  (0.5228, 0.5824) | 0.5128  (0.4838, 0.5418) | 0.5144  (0.4898, 0.5389) | 0.5553  (0.5313, 0.5791) |
| Sensitivity | 0.6955 | 0.6496 | 0.5994 | 0.8324 |
| Specificity | 0.5170 | 0.4786 | 0.4931 | 0.4860 |
| PPV | 0.2647 | 0.2375 | 0.2282 | 0.2882 |

*^a^* Threshold for sepsis prediction (operating point): RETTS red (highest level)

RETTS = Rapid Emergency Triage and Treatment System

n = number of ICU stays with non-missing information at this time point

AUROC = area under the receiver operating characteristic curve

AUPRC = area under the precision-recall curve

CI = confidence interval

PPV = positive predictive value

**Supplemental Table 13: Performance Metrics for Sepsis Alert Predicting Sepsis Up to 3 Hours in Advance, All ICU Stays with Available Data**

| **Sepsis Alert** | 3h | 2h | 1h | 0h |
| --- | --- | --- | --- | --- |
| n | 5430 | 6785 | 13625 | 19770 |
| AUROC | 0.5231 | 0.5188 | 0.5119 | 0.5242 |
| AUPRC | 0.2355 | 0.2290 | 0.2173 | 0.2377 |
| Accuracy *^a^*  ( 95% CI) | 0.7972 (0.7863, 0.8079) | 0.7965 (0.7867, 0.8060) | 0.7929 (0.7860, 0.7997) | 0.798 (0.7924, 0.8036) |
| Sensitivity | 0.0663 | 0.0560 | 0.0437 | 0.0678 |
| Specificity | 0.9800 | 0.9816 | 0.9802 | 0.9806 |
| PPV | 0.4528 | 0.4318 | 0.3552 | 0.4661 |

*^a^* Threshold for sepsis prediction (operating point): Sepsis Alert (“Yes”)

n = number of ICU stays with non-missing information at this time point

AUROC = area under the receiver operating characteristic curve

AUPRC = area under the precision-recall curve

CI = confidence interval

PPV = positive predictive value

**Supplemental Table 14: Performance Metrics for Sepsis Alert Predicting Sepsis Up to 3 Hours in Advance, Population for Algorithm Development and Validation**

| **Sepsis Alert** | 3h | 2h | 1h | 0h |
| --- | --- | --- | --- | --- |
| n | 1095 | 1160 | 1580 | 1700 |
| AUROC | 0.5451 | 0.5329 | 0.5166 | 0.5342 |
| AUPRC | 0.2605 | 0.2411 | 0.2189 | 0.2424 |
| Accuracy *^a^*  ( 95% CI) | 0.7954  (0.7703, 0.8190) | 0.7879  (0.7633, 0.8111) | 0.7791 (0.7578, 0.7994) | 0.7876  (0.7674, 0.8069) |
| Sensitivity | 0.1279 | 0.1078 | 0.0791 | 0.1118 |
| Specificity | 0.9623 | 0.9580 | 0.9541 | 0.9566 |
| PPV | 0.4590 | 0.3906 | 0.3012 | 0.3918 |

*^a^* Threshold for sepsis prediction (operating point): Sepsis Alert (“Yes”)

n = number of ICU stays with non-missing information at this time point

AUROC = area under the receiver operating characteristic curve

AUPRC = area under the precision-recall curve

CI = confidence interval

PPV = positive predictive value

**Supplemental Table 15: Performance Metrics for PRESEP Predicting Sepsis Up to 3 Hours in Advance, All ICU Stays with Available Data**

| **PRESEP** | 3h | 2h | 1h | 0h |
| --- | --- | --- | --- | --- |
| n | 1095 | 1383 | 2864 | 4061 |
| AUROC | 0.6717 | 0.6691 | 0.6657 | 0.6666 |
| AUPRC | 0.3533 | 0.3492 | 0.3466 | 0.3507 |
| Accuracy *^a^*  ( 95% CI) | 0.7861 (0.7750, 0.7969) | 0.7847 (0.7748, 0.7943) | 0.7859 (0.7791, 0.7926) | 0.7867 (0.7810, 0.7923) |
| Sensitivity | 0.2192 | 0.2234 | 0.2168 | 0.2261 |
| Specificity | 0.9279 | 0.9250 | 0.9282 | 0.9269 |
| PPV | 0.4317 | 0.4268 | 0.4301 | 0.4359 |

*^a^* Threshold for sepsis prediction (operating point): PRESEP≥4

PRESEP = Prehospital Early Sepsis Detection score

n = number of ICU stays with non-missing information at this time point

AUROC = area under the receiver operating characteristic curve

AUPRC = area under the precision-recall curve

CI = confidence interval
PPV = positive predictive value

**Supplemental Table 16: Performance Metrics for PRESEP Predicting Sepsis Up to 3 Hours in Advance, Population for Algorithm Development and Validation**

| **PRESEP** | 3h | 2h | 1h | 0h |
| --- | --- | --- | --- | --- |
| n | 1100 | 1170 | 1635 | 1700 |
| AUROC | 0.6130 | 0.6167 | 0.6196 | 0.6339 |
| AUPRC | 0.2688 | 0.2940 | 0.2927 | 0.2868 |
| Accuracy *^a^*  ( 95% CI) | 0.7364  (0.7093, 0.7622) | 0.7419  (0.7158, 0.7667) | 0.7517  (0.7300, 0.7725) | 0.7535  (0.7323, 0.7739) |
| Sensitivity | 0.2227 | 0.2350 | 0.2447 | 0.2559 |
| Specificity | 0.8648 | 0.8986 | 0.8784 | 0.8779 |
| PPV | 0.2917 | 0.3090 | 0.3347 | 0.3439 |

*^a^* Threshold for sepsis prediction (operating point): PRESEP≥4

PRESEP = Prehospital Early Sepsis Detection score

n = number of ICU stays with non-missing information at this time point

AUROC = area under the receiver operating characteristic curve

AUPRC = area under the precision-recall curve

CI = confidence interval

PPV = positive predictive value
